# P-648. Shorter *vs* Standard-Duration Antibiotic Therapy for Nocardiosis: A Multicenter Retrospective Cohort Study

**DOI:** 10.1093/ofid/ofae631.845

**Published:** 2025-01-29

**Authors:** Nofar Hezkelo Attias, Tal schlaeffer, Itay Zahavi, Noga Hasson, Yaara Ben Ari, Basel Darawshe, Idan Levitan, Elad Goldberg, Michal Landes, Vladislav Litchevsky, Haim Ben Zvi, Sharon Amit, Lior Nesher, Jihad Bishara, Mical Paul, Dafna Yahav, Ili Margalit

**Affiliations:** Rabin Medical Center, Petah Tikva, HaMerkaz, Israel; Soroka Medical Center, Beer Sheva, HaDarom, Israel; Rambam Healthcare Campus, Haifa, HaZafon, Israel; Tel Aviv University, Tel Aviv, HaMerkaz, Israel; Lev Hasharon Mental Health Center, Tzur Moshe, HaMerkaz, Israel; Rambam Healthcare Campus, Haifa, HaZafon, Israel; Rabin Medical Center, Petah Tikva, HaMerkaz, Israel; Rabin Medical Center, Petah Tikva, HaMerkaz, Israel; Rabin Medical Center, Petah Tikva, HaMerkaz, Israel; Sheba Medical Center, Ramat Gan, HaMerkaz, Israel; Rabin Medical Center; Tel Aviv University, Petach Tikva, Tel Aviv, Israel; Sheba Medical Center, Ramat Gan, HaMerkaz, Israel; Soroka Medical Center, Beer Sheva, HaDarom, Israel; Rabin Medical Center, Petah Tikva, HaMerkaz, Israel; Rambam Health Care Campus, Haifa, Hefa, Israel; Sheba Medical Center, Ramat Gan, HaMerkaz, Israel; Sheba Medical Center, Ramat Gan, HaMerkaz, Israel

## Abstract

**Background:**

The recommendation to treat nocardiosis for long durations (usually 6-12 months) relies on small case series published decades ago. We implemented real-world data to assess the association between treatment duration and outcomes in nocardiosis.

**Table 1.**
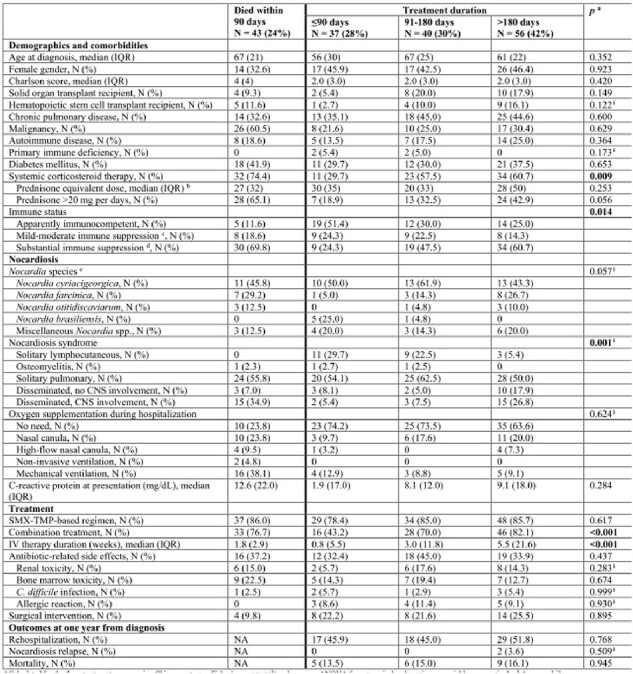
Clinical characteristics and outcomes of individuals diagnosed with nocardiosis, according to treatment duration range a Calculated for the three treatment groups using Chi-square test or Fisher’s exact test (⸙) and one-way ANOVA for categorical and continuous variables, respectively; b Average daily prednisone dosage (in milligrams), during the 90 days prior to presentation. Whenever dexamethasone was used, dosage was converted to the equivalent prednisone dosage; c Individuals with active malignancy, primary immune deficiency, autoimmune diseases, and chronic corticosteroid therapy (≥5 and <20 mg per day); d Solid organ transplant recipients, hematopoietic stem cell transplant recipients, and individuals with acquired immunodeficiency syndrome (AIDS) or on chronic corticosteroid therapy (≥20 mg per day); e Identification to the species level was available for 95/176 (54%) of the cohort. IV = intravenous; NA = not applicable; SMX-TMP = sulfamethoxazole-trimethoprim.

**Methods:**

A multicenter retrospective cohort study comprising individuals with nocardiosis during 2007-2022, excluding those who died within 90 days from diagnosis. Patients were classified into three groups according to treatment duration: short (≤90 days), intermediate (91-180 days), and long ( >180 days). We compared overall mortality, relapse rates, and antibiotic-related adverse events at 1-year. Treatment duration was cross tabulated with immune status and nocardiosis syndrome, considering that clinical judgment has led physicians to deviate from recommended long durations in appropriate cases.

**Figure 1.**
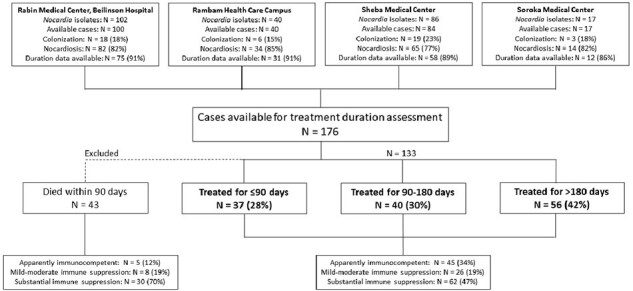
Study flow diagram

**Results:**

A total of 176 individuals were diagnosed with nocardiosis during the study period, 43 (24%) of whom died within 90 days and were excluded (Figure 1). Of the remaining 133 individuals, 45 (34%) were apparently immunocompetent, 26 (19%) and 62 (47%) had mild-moderate and substantial immune suppression, respectively. Patients received short (N=37, 28%), intermediate (N=40, 30%) and long (N=56, 42%) treatment durations. Longer treatment duration was positively associated with systemic corticosteroid use (*p*=0.009), immune suppression (*p*=0.014), and nocardiosis syndrome (longer durations for dissemination, *p*=0.001; Table, Figure 2). By 1-year, 20 (15%) individuals died and 2 (1.5%) relapsed. Treatment duration was not associated with mortality (*p*=0.977; Figure 3) or nocardiosis relapse (*p*=0.509). Of those with solitary pulmonary nocardiosis (N=73), none of the 20 immunocompetent individuals died by 1-year, while 5/16 (31%), and 6/37 (17%) of those with mild-moderate and substantial immune suppression, respectively, died, regardless of treatment duration. Adverse events rate did not differ significantly between duration groups.

**Figure 2.**
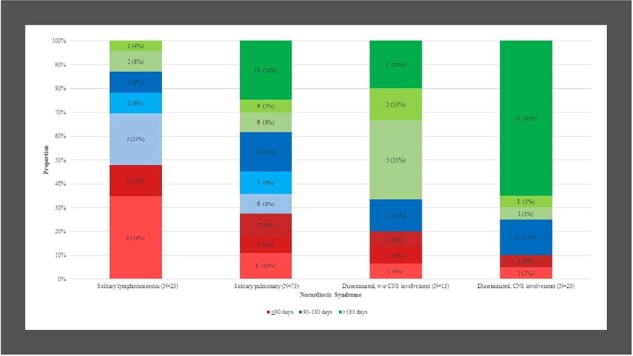
Distribution of treatment duration groups according to nocardiosis clinical syndrome and immune status

For each color, shades signify immune status (apparently immunocompetent individuals, and those with mild-moderate or substantial immune suppression): darker shades signify diminished immunity.

**Conclusion:**

With clinically guided case-by-case patient selection nocardiosis can be safely treated for durations significantly shorter than traditionally recommended.

**Figure 3.**
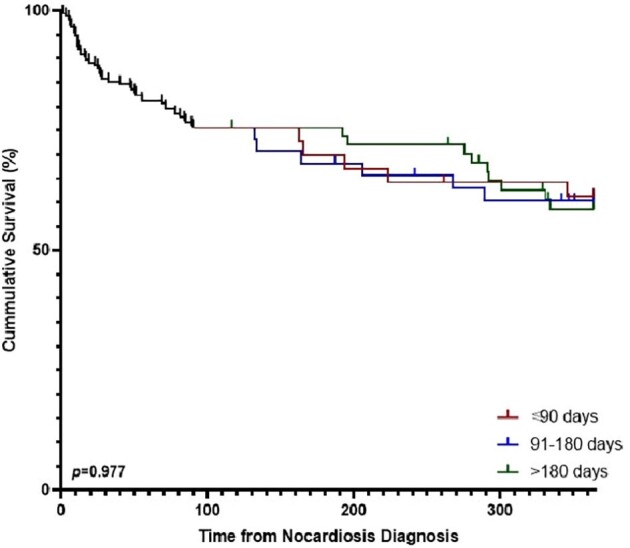
Survival curves of the entire cohort, according to treatment group

The entire cohort is presented as a single curve up to 90 days, then divided into 3 treatment groups, according to the total treatment duration. p value calculated using Log-rank test.

**Disclosures:**

**All Authors**: No reported disclosures

